# Focusing on Solid Partnerships Across Multiple Sectors for Population Health Improvement

**Published:** 2010-10-15

**Authors:** Stephanie B. Coursey Bailey

**Affiliations:** Office of the Chief of Public Health Practice, Centers for Disease Control and Prevention

## Introduction

Partnerships create a way forward when no clear solution exists and no single entity can claim the necessary expertise, authority, or resources to bring about change. Cross-sectoral partnerships are needed to mobilize community action and improve population health.

The Mobilizing Action Toward Community Health (MATCH) articles in this issue of *Preventing Chronic Disease* reveal compelling themes, issues, and recommendations for improving population health. These include many challenges, such as how to scale up successful partnership efforts (1,2), determine if and how partnership activity can be correlated with changing health metrics (1-5), expand the use of incentives for improvement (1,3,4,6), and strengthen groups' distributive leadership and governance (1,2,4-6).

## Building Blocks for Effective Multisectoral Partnerships

The MATCH articles identify characteristics that are needed to build and sustain successful partnerships: 1) social value, 2) common goals, 3) rewards and incentives, and 4) comprehensive and coordinated approaches.

According to Wei-Skillern, the driving force of social entrepreneurship is the creation of social value rather than personal or shareholder wealth (1). She describes a form of networking that leverages organizational resources and expertise to achieve greater social impact. The network approach does not necessarily require more resources; rather, the goal is to make the best use of existing resources.

Fawcett et al assert that systems require interconnectedness to support effective and sustained efforts to change conditions (7). Having common goals helps create a unified sense of mission and encourages collective engagement to improve community health. This is best realized if a comprehensive and coordinated framework is adopted, such as the 2002 Institute of Medicine (IOM) framework for collaborative public health action in communities (8). The IOM framework outlines 12 collaborative processes that can facilitate change and improvement in population-level outcomes.

### Lessons from the Healthy Communities movement

Pittman discusses some consistent patterns and themes of the Healthy Communities movement: strong distributed leadership and governance, existence of a health status improvement focus that distributes the broad-focused community intervention into its various and targeted parts, metrics to help guide the local efforts, accountable leadership, well-supported infrastructure, and an investment in data systems that integrate across efforts (2). This movement lays the foundation for what the European Union has adopted as health in all policies, which shifts the emphasis from individual lifestyles and single diseases to societal factors and actions that shape our everyday living environments. This approach serves as a motivator for all available measures in all policy fields.

### The call to build a new generation of intersectoral partnerships

Mays asserts that large-scale implementation partnerships affecting communities most at risk remain rare in practice (4). The paucity of this type of partnership may be because of the nature and constraints of public and private funding mechanisms. Funds are usually allocated for a limited time and come with many regulations. There is often not enough money to go beyond the pilot. Pilot projects too often remain just that. Moving to implementation requires broad support, proven value, and additional resources.

### Incentives for the business community

Workforce health, the community's health, and metrics that are appropriate for businesses can foster business sector engagement in population health. We may be at the cusp of a paradigm shift as business leaders become aware of the cost savings associated with a healthy workforce. If business leaders understand the close relationship between employee health and community environments, the decision to be involved in population health improvement is an easy one. Many examples exist of businesses participating in initiatives to strengthen community health and developing internal workplace initiatives on their own. As Webber and Mercure acknowledge, people operating from a business mindset may not internalize the value or relevance of typical population health measures (5). However, metrics (such as the burden of disease) can influence business decisions, such as where to locate a business.

### Leadership, governance, and standards

Partnerships can and should be viewed as social networks in which breadth, density, and organizational centrality are features that influence performance. Other characteristics include clear goals, effective leaders who see beyond the boundaries of their organizations, accountability, and a well-supported infrastructure.

There is a potential economic basis for governance that promotes well-being in a country or region. Fox suggests that governance could be strengthened by creating and according political protection to public organizations (3).

Performance and accreditation standards for government public health agencies represent opportunities for strengthening incentives for partnerships. For 3 years, 2005-2007, approximately 750 communities used Mobilizing for Action through Planning and Partnerships to conduct community assessments and develop partnerships (9). Additional promising models should be developed and tested, such as the state of Vermont's Community Based Payment Reform (6).

## The Difficulty of Determining Direct Correlation or Causation

From a research perspective, isolating the effects of partnerships on community-level health behaviors remains a challenge. Better systems are needed for measuring and reporting what happens in a community. Communities and programs evolve over time, including changes in leadership, participants, levels of participation, and environmental contexts. These complex and dynamic variables and circumstances limit the degree to which rigorous evaluation may be applied to partnership structure, function, and achievement. The value of metrics in guiding local efforts, providing a form of accountability and transparency, and creating a constituency for local political support and policy change is not lost on communities. An integrative data system would help researchers to measure the effect and effectiveness of multisectoral policies and intervention.

Ultimately, health outcomes should be the measure on which any health intervention is judged. However, the patience and commitment required to improve population health outcomes over the long term run counter to our strong cultural desire for instant answers and immediate gratification. Such a system, based only on short-term change, is incompatible with the provision of meaningful incentives for population health improvement. Going forward, systems must be developed and institutionalized to reward the longer term upstream solutions.

## Conclusion

This group of articles provides diverse perspectives on partnerships for population health improvement. In considering them, the following recommendations emerge for research and practice:

1. Invest in data systems that can better integrate the multiple sources of data affecting population health.
2. Develop incentives for policy actions and leadership while blunting disincentives for participation.
3. Adopt a network mindset to overcome the seemingly intractable barriers to achieving population health. This involves creating social value and having common goals.
4. Create opportunities for cross-sector networking and collaboration to build relationships between and among leaders.
5. Develop and advocate for sustained funding mechanisms as opposed to short-term grants.
6. Establish metrics to inform and motivate cross-sectoral action — with emphasis on including partnerships with the business community.

Partnerships for population health improvement help us make better use of existing resources, and they expand the dialogue to businesses, faith-based organizations, education, commerce, public safety, housing, transportation, decision makers, and community members. However, in the context of this young discipline of population health, many questions on partnerships require further exploration. These include questions that relate to organizational partnerships, costs, leadership characteristics, and community dynamics.

Implementing the recommendations would likely have unintended consequences. Recognizing health in all policies could lead, for example, to increased competition for finite resources across sectors. However, potential benefits for community health justify both the risk and the effort.
